# Frequency of coronavirus disease 2019 (COVID-19) symptoms in healthcare workers in a large health system

**DOI:** 10.1017/ice.2020.1297

**Published:** 2020-10-26

**Authors:** Jason H. Malenfant, Caitlin N. Newhouse, Alice A. Kuo

**Affiliations:** 1 Public Health & Preventive Medicine Program, David Geffen School of Medicine, University of California, Los Angeles (UCLA), Los Angeles, California; 2 Division of Pediatric Infectious Diseases, David Geffen School of Medicine, University of California, Los Angeles (UCLA), Los Angeles, California; 3 Division of Medicine-Pediatrics, David Geffen School of Medicine, University of California, Los Angeles (UCLA), Los Angeles, California

*To the Editor*—Since the early phases of the coronavirus disease 2019 (COVID-19) epidemic across the United States, identifying and tracking healthcare worker (HCW)-to-HCW transmission has been a major priority given the risk of exposing other colleagues, exposing vulnerable patients, and issues of limited staffing. Policy guidance regarding symptomatic screening for exclusion from work to mitigate transmission has not evolved much over the course of the pandemic to date.

From March 20 through April 10, 2020, a total of 2,193 severe acute respiratory coronavirus virus 2 (SARS-CoV-2) tests were ordered for HCWs in our institution, and 174 HCWs in our health system tested positive (8% positivity rate). Symptoms were not recorded for 3 individuals. Of the remaining 171 HCWs included in our symptom analysis, 69 (40%) were registered nurses, certified nursing assistants, or care partners; 31 (18%) were clinical administrative employees; 25 (15%) were physicians (of whom 11 were house staff); and 46 (27%) consisted of respiratory therapists, radiology technicians, custodial services, or other ancillary service delivery workers. In total, 119 HCWs (70%) worked in the inpatient setting, 41 (24%) worked in ambulatory clinics, and 11 (6%) had their primary workplace at an offsite office (eg, a telehealth call center).

The most common initial symptoms were cough (51%), fever (41%), and myalgia (38%). Additional common symptoms included headache (30%), nasal congestion and/or runny nose (28%), severe fatigue (26%), and sore or scratchy throat (25%). Loss of smell (16%) and loss of taste (15%) were also reported (Table 1). Variability was noted in the number of initial symptoms: 19% endorsed only 1 presenting symptom, 23% had 2 initial symptoms, 26% had 3 symptoms, and 33% had 4 symptoms or more.

**Table 1.** Initial Signs and Symptoms Reported in HCWs Diagnosed With COVID-19

Signs and Symptoms^a^	HCWs (n=171), No. (%)
Cough	88 (51)
Fever	70 (41)
Myalgia	65 (38)
Headache	52 (30)
Nasal congestion/runny nose	48 (28)
Severe fatigue	44 (26)
Sore throat	42 (25)
Loss of smell	28 (16)
Loss of taste	26 (15)
Chills	24 (14)
Difficulty breathing	18 (11)
Chest tightness/pain	13 (8)
Diarrhea	12 (7)
Loss of appetite	9 (5)
Vomiting	2 (1)

Note. HCWs, healthcare workers.

a HCWs could report >1 sign/symptom.

Of those HCWs with only 1 initial symptom, the most common complaint was cough (47%), followed by a sore or scratchy throat (19%). Only 3 of the 32 HCWs with 1 initial symptom (9%) reported fever. Only 1 person (3%) reported loss of smell as their only initial symptom. Of those HCWs reporting 2 initial symptoms, the most common combination of initial symptoms was fever and cough (15%), followed by headache and myalgia (10%). Approximately one-third of all HCWs did not report fever or cough as an initial presenting symptom.

Almost half (49%) of all HCWs surveyed reported working at least 1 day while symptomatic and before calling the HCW hotline to report symptoms. Of these, 57% worked for 1 day, 18% for 2 days, 10% for 3 days, and 15% for 4 or more days (maximum, 6 days).

Early reports from around the world described COVID-19 as an illness characterized primarily by fever and cough.^1,2^ Due to limited testing capacity, testing access was often initially limited to individuals who presented with these symptoms. Because of the potential for HCWs to spread SARS-CoV-2 to patients and coworkers, our health system opted for a low threshold for testing to better characterize the spectrum of disease and reduce inadvertent spread. Our data suggest that SARS-CoV-2 infection can present with a wider variety of mild symptoms than was suggested by early studies. In fact, one-third of HCWs in our study did not report fever or cough as one of their symptoms, and these individuals would have been missed by more restrictive testing guidelines. Additionally, almost half (49%) of the HCWs continued to work while experiencing symptoms, some for several days. There are several possible explanations for this. For example, it was early in the pandemic and the mild presentation of COVID-19 was not widely recognized.

Our findings have important implications for healthcare systems and other employers regarding when to test employees and when to implement mandatory stay-home-from-work policies. Employee health programs should message that early COVID-19 can present with subtle viral symptoms, including those mimicking mild upper-respiratory infections or allergies, and that individuals should have a low threshold to present for evaluation and testing.

Additionally, our data highlight the importance of universal symptom monitoring through either a daily survey or mandatory entry point at facility entrances. Screening HCWs solely for fever would have missed 60% of SARS-CoV-2–positive individuals in our sample. Health systems and other work places should consider strategies that screen for a variety of signs and symptoms of early COVID-19 infection. Universal symptom monitoring can help create a culture change in the work place, which includes being attuned to subtle changes in health and staying home from work for mild illness. This may be a difficult culture shift for various reasons including financial incentives to continue working, historical emphasis on presenteeism, and reluctance to miss work and call in a coworker for mild symptoms that may not impede one’s ability to work. However, with the COVID-19 pandemic, this critical shift is essential to stop potential transmission to vulnerable patients and other HCWs.

Health systems, school districts, and other work places can learn from this experience to develop pragmatic and effective infection control policies in the era of COVID-19. Often, areas for improvement in infection prevention can be identified to help mitigate the spread of SARS-CoV-2 within a critical work force. Especially with concern of a second and potentially multiple further waves of COVID-19, leaders can use these lessons to develop better strategies, further preparedness, and reduce burdens on our hospital systems and labor force.

## References

[1] Zhang JJ , Dong X , Cao YY , et al. Clinical characteristics of 140 patients infected by SARS-CoV-2 in Wuhan, China. Allergy 2020;75:1730–1741. 32077115 10.1111/all.14238

[2] Chen N , Zhou M , Dong X , et al. Epidemiological and clinical characteristics of 99 cases of 2019 novel coronavirus pneumonia in Wuhan, China: a descriptive study. Lancet 2020;395:507–513. 32007143 10.1016/S0140-6736(20)30211-7 PMC7135076

